# Compensatory regulation of Na^+^ absorption by Na^+^/H^+^ exchanger and Na^+^-Cl^-^ cotransporter in zebrafish (*Danio rerio*)

**DOI:** 10.1186/1742-9994-10-46

**Published:** 2013-08-07

**Authors:** Wei-Jen Chang, Yi-Fang Wang, Huei-Jyun Hu, Jung-Hsuan Wang, Tsung-Han Lee, Pung-Pung Hwang

**Affiliations:** 1 Institute of Cellular and Organismic Biology, Academia Sinica, Taipei, Taiwan; 2 Molecular and Biological Agricultural Science Program, Taiwan International Graduate Program, Academia Sinica, Taipei, Taiwan; 3 Graduate Institute of Biotechnology and Department of Life Science, National Chung-Hsing University, Taichung, Taiwan; 4 Institute of Fishery Science, National Taiwan University, Taipei, Taiwan; 5 Institute of Zoology, National Taiwan University, Taipei, Taiwan

**Keywords:** Na^+^-Cl^-^ cotranspoter, Na^+^/H^+^ exchanger, Ionocyte, Acid acclimation

## Abstract

**Introduction:**

In mammals, internal Na^+^ homeostasis is maintained through Na^+^ reabsorption via a variety of Na^+^ transport proteins with mutually compensating functions, which are expressed in different segments of the nephrons. In zebrafish, Na^+^ homeostasis is achieved mainly through the skin/gill ionocytes, namely Na^+^/H^+^ exchanger (NHE3b)-expressing H^+^-ATPase rich (HR) cells and Na^+^-Cl^-^ cotransporter (NCC)-expressing NCC cells, which are functionally homologous to mammalian proximal and distal convoluted tubular cells, respectively. The present study aimed to investigate whether or not the functions of HR and NCC ionocytes are differentially regulated to compensate for disruptions of internal Na^+^ homeostasis and if the cell differentiation of the ionocytes is involved in this regulation pathway.

**Results:**

Translational knockdown of *ncc* caused an increase in HR cell number and a resulting augmentation of Na^+^ uptake in zebrafish larvae, while NHE3b loss-of-function caused an increase in NCC cell number with a concomitant recovery of Na^+^ absorption. Environmental acid stress suppressed *nhe3b* expression in HR cells and decreased Na^+^ content, which was followed by up-regulation of NCC cells accompanied by recovery of Na^+^ content. Moreover, knockdown of *ncc* resulted in a significant decrease of Na^+^ content in acid-acclimated zebrafish.

**Conclusions:**

These results provide evidence that HR and NCC cells exhibit functional redundancy in Na^+^ absorption, similar to the regulatory mechanisms in mammalian kidney, and suggest this functional redundancy is a critical strategy used by zebrafish to survive in a harsh environment that disturbs body fluid Na^+^ homeostasis.

## Introduction

Sodium is the dominant cation in vertebrate body fluids, and therefore sodium regulation is one of most important tasks in maintaining body fluid homeostasis in vertebrates. In mammalian kidney, about two-thirds of sodium reabsorption occurs in the proximal tubule, and is achieved mainly by apically-expressed Na^+^/H^+^ exchangers (NHE). The remaining sodium is reabsorbed by the distal convoluted tubules and the collecting ducts, via Na^+^-Cl^-^ cotransporters (NCC) and epithelial sodium channels (ENaC), respectively [1]. Hormonal control of sodium reabsorption occurs primarily in the distal convoluted tubules and the collecting ducts [2], suggesting that while NHE performs the majority of sodium reabsorption, this process is fine-tuned by NCC and ENaC in the mammalian kidney. Sodium transport along the nephron can be regulated through controlling the abundance, activity, and subcellular distribution of transporters [3]. Previous studies have demonstrated that a high-salt diet provokes subcellular redistribution and decreased abundance of NCC, but does not affect the abundance or activity of NHE3 [3-5]. In addition, the hormones angiotensin II and aldosterone, which are the major factors that determine the sodium reabsorption rate, positively regulate NCC activity via a WNK4 (with-no-lysine kinase 4)-dependent pathway [6,7]. Taken together, dietary salt change, activates the functional regulation of NCC in mammalian kidney through an aldosterone-dependent and WNK4-mediated mechanism [8].

Sodium transporters expressed along the nephron are able to compensate for each other in sodium absorption mechanisms. In *nhe3* knockout mice, the levels of sodium-phosphate cotransporter (NaPi2) and the 70 kDa ENaC γ-subunit were found to be increased [9]. Moreover, the abundance of the 70 kDa ENaC γ-subunit was also reported to be increased in *ncc* knockout mice [9]. Loss of NCC activity in mice resulted in only a subtle perturbation of Na^+^ homeostasis, which was presumed to arise from a compensatory increase in ENaC activity [10,11]. Compensation through differential regulation of multiple Na^+^ uptake pathways is essential for internal Na^+^ homeostasis in mammals, and from an evolutionary physiological perspective, it may be conserved in other osmoregulatory vertebrates. However, there is little available information on this process in other vertebrates.

Teleosts, as an aquatic vertebrate, maintain body fluid homeostasis of Na^+^ (and other ions) through active absorption of this ion from freshwater (FW) environments with Na^+^ levels less than 1 mM. The Na^+^ uptake mechanisms in fish, mainly conducted in the gills or skin (during embryonic stages), are analogous to those in mammalian kidney, in terms of transporter expression and function in the ionocytes [12-15]. Zebrafish has recently proved to be a competent model for molecular physiological studies on ion- and osmo-regulation in fish, because of its advantages in terms of gene manipulation and availability of genome databases [15-18]. According to the recently proposed zebrafish ion regulation model, there are four subtypes of ionocyte, as follows: Na^+^-K^+^-ATPase-rich (NaR) cells, H^+^-ATPase-rich (HR) cells, Na^+^-Cl^-^ cotransporter (NCC)-expressing cells, and K^+^-secreting (KS) cells; these have been identified to express distinct sets of ion transporters, and thus to conduct different ions [15,18,19]. Of these ionocytes, HR cells, which co-express NHE3b and H^+^-ATPase in the apical membrane, were identified to be the major cell type responsible for Na^+^ absorption and H^+^ secretion in zebrafish [20-23]. On the other hand, NCC cells, which apically express NCC, play a minor role in Na^+^ uptake in zebrafish [24]; this was demonstrated by two lines of evidence: sodium green, an Na^+^ fluorescence probe, accumulates only in HR cells, and metolazone, an NCC-specific inhibitor, does not impair sodium green accumulation in HR cells [21].

In zebrafish HR cells, certain co-expressed transporters and enzymes (vacuolar H^+^-ATPase, ammonia transporter (Rhcg1), 2 carbonic anhydrase (CA2-like and CA15a), and anion exchanger (AE1b)) were found to affect the activity and function of NHE3b [22,25-29]. The activity of electroneutral NHE3b in the apical membrane of zebrafish HR cells depends on the gradients of Na^+^ and H^+^ across the cell membrane [30], and therefore acidic FW environments do not favor NHE3b activity. Exposure to pH 4 FW was found to suppress *nhe3b* mRNA expression in the zebrafish gill [23]. Notably, the same acid treatment was reported to cause an initial decrease in body Na^+^ content, and thereafter a recovery of content accompanied by increased Na^+^ uptake [31]. This raises the possibility that an alternative pathway, not involving NHE3b, may play a compensatory role in Na^+^ homeostasis in zebrafish under environmental acid stress.

An ortholog of mammalian NCC, zSlc12a10.2, was reported to be specifically expressed in an ionocyte type distinct from NHE3-expressing HR-type cells in zebrafish skin/gills; this NCC-type ionocyte was subjected to functional analysis, revealing the presence of an apical Na^+^ and Cl^-^ uptake pathway [24]. In addition, expression of *nhe3b* and *ca15a* was found to be up-regulated in NCC morphants [24]. Based on these results, we hypothesized that Na^+^ absorption in zebrafish skin/gill is similar to that in mammalian kidneys; NHE3 performs the majority of Na^+^ uptake, and the other transporters, such as NCC, play a supplementary role; furthermore, we hypothesized that these Na^+^ transport proteins would be able to compensate for one another in the regulation of body fluid Na^+^ homeostasis.

Herein, we tested the hypothesis that compensatory regulation of Na^+^ uptake mechanisms is conserved in teleosts. The experiments were designed to determine whether *ncc* expression is up-regulated when *nhe3b* expression is decreased (and vice versa), and whether NCC is required for Na^+^ absorption in acid-acclimated zebrafish.

## Results

### NCC loss-of-function induces differentiation of HR cells

**Figure 1:**
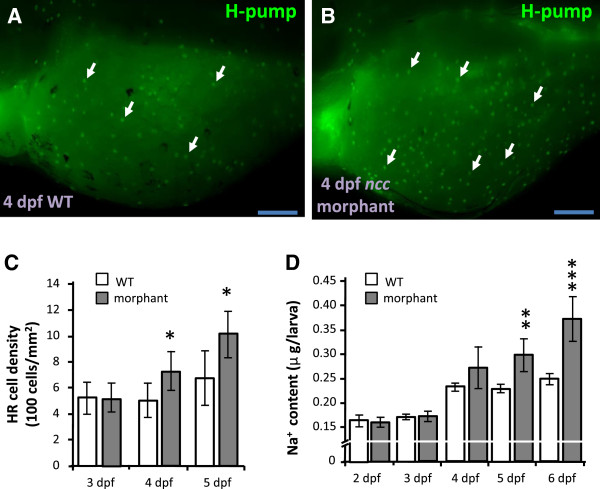
**Knockdown of *ncc *increases HR cell number and Na**^**+ **^**accumulation. (A, B)**: H^+^-ATPase (H-pump) immunostaining signals (arrow) in 4-dpf wild type larvae (WT) and *ncc* morphants. Scale bar = 100 μm. **(C, D)**: Comparisons of HR cell density **(C)** and whole body Na^+^ content **(D)** between wild type larvae (white bars) and *ncc* morphants (gray bars) at different developmental stages (n = 8 in *C* and 6 in *D*). Mean ± SD. *p < 0.05; **p < 0.01; ***p < 0.001 (Student’s *t*-test).

Compensatory expression of Na^+^ transporters was previously observed in NCC null mice [9]. In order to identify whether similar compensation occurs in zebrafish, the development of HR cells was observed in *ncc* morphants. As shown in Figure 1A-C, knockdown of NCC translation enhanced differentiation of HR cells in 4- and 5 dpf larvae. The Na^+^ content remained at wild type levels in 2- and 3 dpf morphants, but was significantly increased in 5- and 6 dpf larvae (Figure 1D). Initially, NCC loss-of-function does not significantly affect Na^+^ accumulation, but by later stages, Na^+^ accumulation is enhanced. This may be due to an accompanying increase of HR cells, which express the major Na^+^-uptake transporter, NHE3b. This suggests that an increase of HR cells (and thus NHE3b expression) may compensate for the loss of NCC in terms of Na^+^ uptake.

### Knockdown of *gcm2* results in increased NCC cell differentiation and subsequent recovery of Na^+^ content

**Figure 2:**
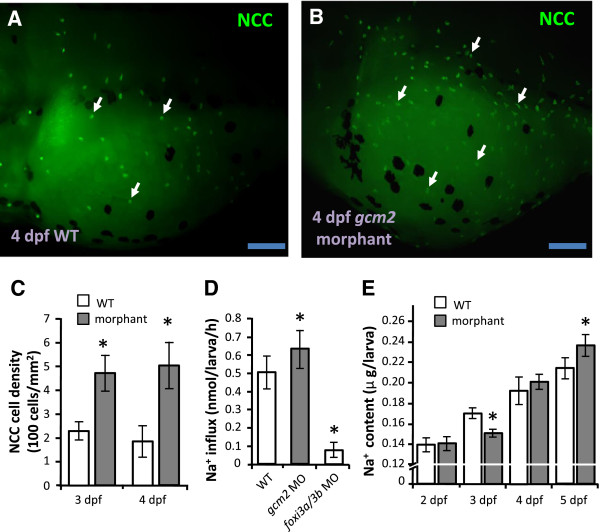
**Loss of HR cells by *gcm2 *knockdown increases NCC cell number and Na**^**+ **^**absorption ability. (A, B)**: Immunostaining images of NCC cells (arrow) in 4-dpf wild type larvae (WT) and *gcm2* morphants. Scale bar = 100 μm. **(C)**: Comparison of NCC cell density between wild type larvae (white bars) and *gcm2* morphants (gray bars) at different developmental stages (*n* = 8). **(D)**: Na^+^ influx in 4-dpf wild types, *gcm2* morphants, and *foxi3a*/*3b* morphants (*n* = 6). **(E)**: Whole body Na^+^ content in wild type larvae (white bars) and *gcm2* morphants (gray bars) at different developmental stages (*n* = 6). Mean ± SD. *p < 0.05 (Student’s *t*-test).

The transcription factor Gcm2 is known to control the differentiation of HR cells, which express NHE3b [23,32,33]. Blocking HR cell differentiation with *gcm2* MOs resulted in an increase of NCC cells (Figure 2A-C). This increase in NCC cells may be related to an increase in the ability of morphants to absorb Na^+^. Compared to 4 dpf wild type larvae, Na^+^ influx was significantly increased in *gcm2* morphants, but was dramatically decreased in *foxi3a*/*3b* morphants (Figure 2D). The transcription factors Foxi3a and Foxi3b control the differentiation of NaR and HR cells [34,35], and were also found to be required for the differentiation of NCC cells (Additional file 1: Figure S1). Closer inspection of the *gcm2* morphants revealed an initial decrease of Na^+^ content at 3 dpf, which was restored to wild type levels at 4 dpf; by 5 dpf, the Na^+^ content of the morphants had exceeded that of the wild type (Figure 2E). Taken together, it appears that loss of HR cells impairs the Na^+^ uptake function, and subsequently stimulates NCC cell differentiation resulting in a compensatory increase in Na^+^ accumulation.

### Knockdown of *nhe3b* results in increased NCC cell differentiation and subsequent recovery of Na^+^ content

**Figure 3:**
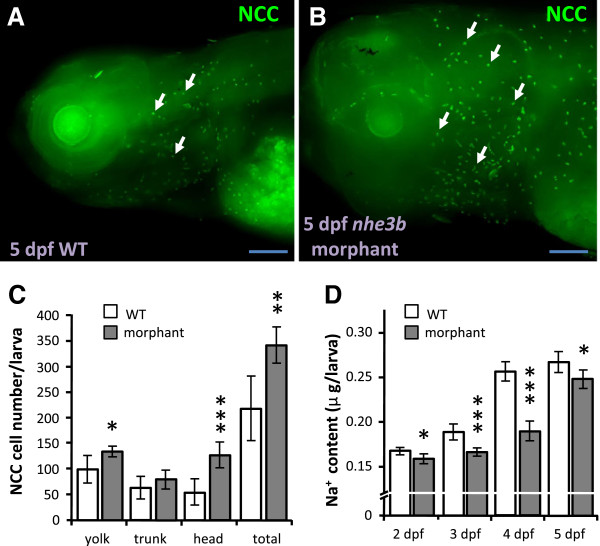
**Knockdown of *nhe3b *increases NCC cell number. (A, B)**: NCC (arrow) immunostaining images of 5-dpf wild type larvae (WT) and *nhe3b* morphants. Scale bar = 100 μm. **(C)**: Comparison of NCC cell number in different areas between 5-dpf wild type larvae (white bars) and *nhe3b* morphants (gray bars) (*n* = 8). **(D)**: Whole body Na^+^ content in wild type larvae (white bars) and *nhe3b* morphants (gray bars) at different developmental stages (*n* = 6). Mean ± SD. *p < 0.05; **p < 0.01; ***p < 0.001 (Student’s *t*-test).

A similar compensatory effect was also observed in *nhe3b* morphants. NCC cells were increased in 5-dpf *nhe3b* morphants as compared to wild type larvae, and the increase was mainly observed in the head region of the morphants (Figure 3A-C). Knockdown of *nhe3b* significantly decreased Na^+^ accumulation in the larvae from as early as 2 dpf, but levels had begun to recover by 5 dpf (Figure 3D). As for *gcm2* knockdown, down-regulation of *nhe3b* translation impairs Na^+^ uptake, which is thereafter compensated by enhanced NCC cell differentiation and Na^+^ absorption ability.

### Expression of *nhe3b* in adult gills is inhibited by acidic environments

**Figure 4:**
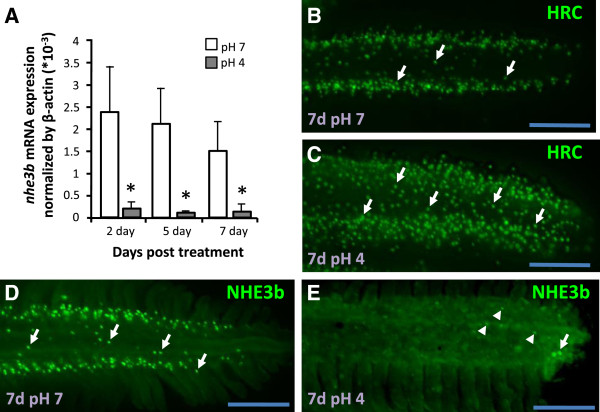
**Down-regulation of *nhe3b *in gills under acid stress. (A)**: Expression of *nhe3b* mRNA in gills treated with control pH7 FW (white bars) or acidic pH4 FW (gray bars) for the indicated days. Mean ± SD (*n* = 6). *p < 0.05 (Student’s *t*-test). **(B-E)**: Immunostaining images of H^+^-ATPase (H-pump) and NHE3b in gills from adult zebrafish maintained in pH7 FW **(B, D)** or pH4 FW **(C, E)** for 7 d. Arrow, NCC or NHE3b signal; arrow head, weaker NHE3b signal. Scale bar = 100 μm.

Next, we investigated compensation of Na^+^ uptake function during acclimation to an acidic environment. The majority of *nhe3b* mRNA expression in adult gills was suppressed during acclimation to an acidic environment (Figure 4A). Immunostaining revealed that HR cells were markedly increased, while NHE3b signals were significantly decreased, after acid acclimation (Figure 4B-E). Therefore, *nhe3b* mRNA and NHE3b protein are both decreased in zebrafish gills after acid treatment.

### NCC cell differentiation in adult gills is up-regulated after acid acclimation

**Figure 5:**
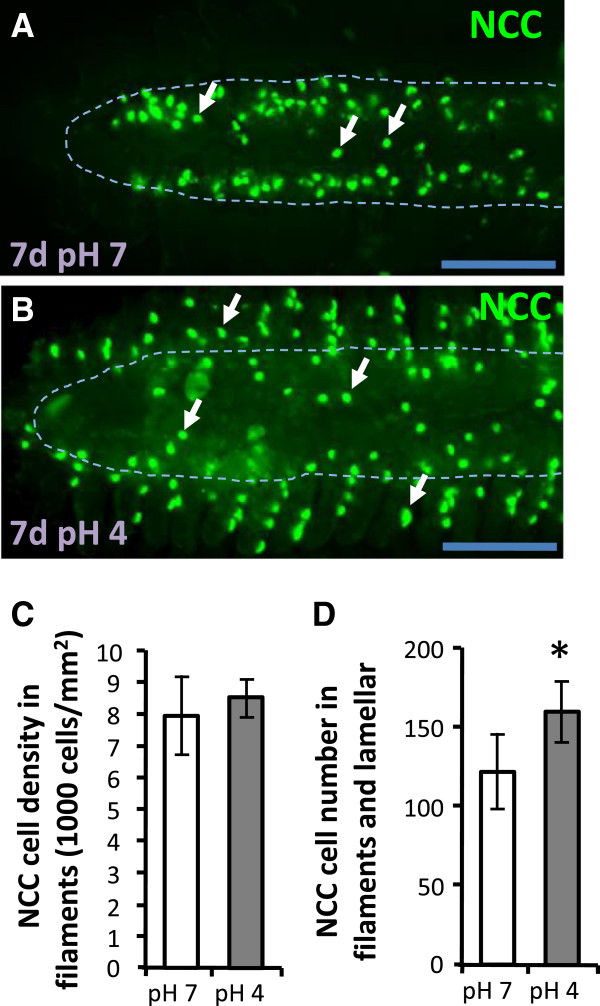
**NCC is up-regulated in gills under acid stress. (A, B)**: Immunostaining images of NCC (arrow) in gills filaments under pH7 or pH4 FW for 7d. **(C, D)**: Comparisons of NCC cell density in gill filaments **(*C*)** and of NCC cell number in both gill lamella and filament **(*D*)** between pH7 (white bars) and pH4 FW (gray bars) for 7d. Means ± SD (*n* = 8). *p < 0.05 (Student’s *t*-test). Scale bar = 100 μm.

The NCC cell density in gill filaments was not changed after acid acclimation for 7 d (Figure 5A-C); however, the total number of NCC cells in both the filaments and lamellae was increased (Figure 5A-B, E). In summary, NHE3b is down-regulated and NCC is up-regulated in adult gills during acclimation to acidic environments.

### Acid acclimation of larvae results in increased NCC cell differentiation and a gradual recovery of Na^+^ content

**Figure 6:**
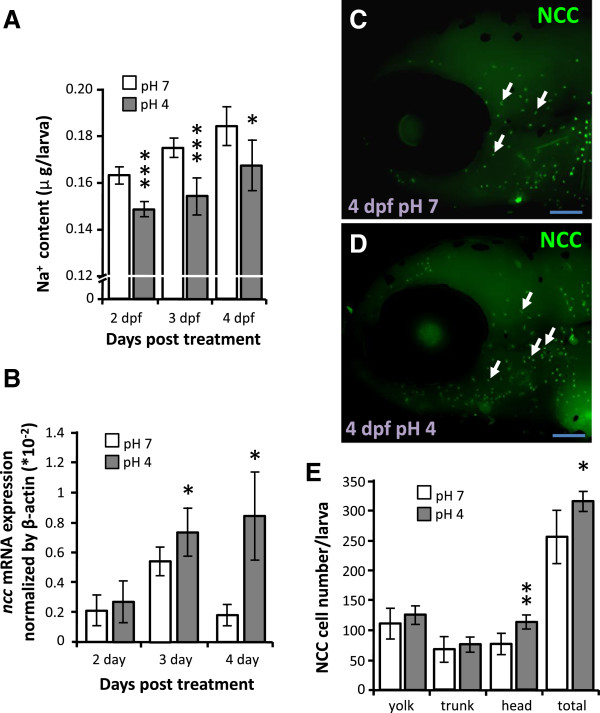
**Effects of acid stress on Na**^**+ **^**accumulation and NCC expression in zebrafish larvae. (A, B)**: Comparison of whole body Na^+^ content **(A)** and *ncc* mRNA expression **(B)** in larvae treated with control pH7 (white bars) or acidic pH4 FW (gray bars) for 4 d. Mean ± SD (*n* = 6). **(C, D)**: Immunostaining images of NCC (arrow) in 4-dpf larvae under pH7 or pH4 FW for 4 d. Scale bar = 100 μm. **(E)**: Comparison of NCC cell number in different areas of 4-dpf larvae treated with pH7 (white bars) or pH4 FW (gray bars) (*n* = 8). *p < 0.05; **p < 0.01; ***p < 0.001 (Student’s *t*-test).

Whole body Na^+^ content was significantly decreased in acid-acclimated larvae as compared to controls raised in pH 7 FW during the first 2~3 d (Figure 6A); however, the difference between test and control groups declined by 4 dpf, suggesting a recovery of Na^+^ absorption ability in the acid-acclimated larvae. In addition, expression of *ncc* mRNA in larvae was significantly increased after 3~4 d acid treatment (Figure 6B), and NCC cell number in the head region, and thus larval skin overall, was also increased after acid acclimation (Figure 6C-E). This suggests that the recovery of Na^+^ accumulation may have resulted from increased *ncc* expression and NCC cell differentiation during acid acclimation.

### NCC is required for compensatory regulation of Na^+^ uptake under acid stress

**Figure 7:**
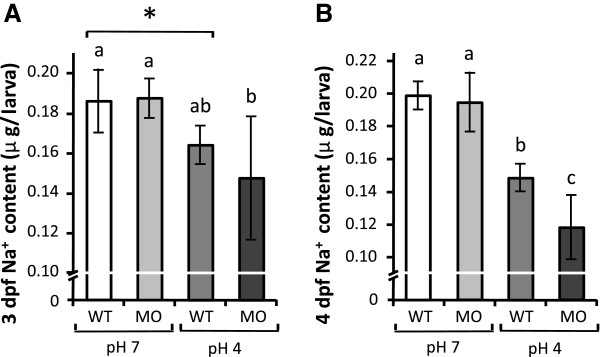
**Effect of *ncc *knockdown on Na**^**+ **^**accumulation in zebrafish larvae under acid stress. (A, B)**: Whole body Na^+^ content in wild type (WT) larvae and *ncc* morphants (MO) treated with control pH7 or acidic pH4 FW for 3 d **(A)** or 4 d **(B)**. Mean ± SD (*n* = 6). *p < 0.05 (Student’s *t*-test). ^abc^significant difference (One-way ANOVA, Tukey’s multiple-comparison).

The whole body Na^+^ contents of wild type larvae and *ncc* morphants were compared after treatment with pH 7 (control) or pH 4 FW. The Na^+^ contents of wild type and *ncc* morphants maintained in pH 7 FW were not significantly different (Figure 7A, B). However, the Na^+^ content in 3 dpf *ncc* morphants was lower (albeit not significantly) than that in wild type larvae upon acid stress; this decrease was greater and significant at 4 dpf (Figure 7A, B), suggesting that NCC is necessary for the compensatory regulation of Na^+^ absorption during acid acclimation.

## Discussion

Like the mammalian kidney, zebrafish skin and gills have been proposed to perform Na^+^ uptake via a variety of Na^+^ transporters, including ENaC, NHE3 and NCC [14-16,36]. Certain pharmacological experiments have suggested a role for ENaC in Na^+^ uptake mechanisms in zebrafish [21]; however, a sequence homologous to ENaC has not yet been identified in zebrafish [14,15]. Yan et al. [23] identified NHE3b in HR cells, and analyzed changes in *nhe3b* mRNA expression induced by low-Na^+^ FW in the cells. Esaki et al. [21] demonstrated that treatment with EIPA, an NHE inhibitor, blocked both sodium green (a fluorescent Na^+^ probe) accumulation and ^24^Na^+^ influx in zebrafish larvae [21], suggesting that NHE3 is a major transporter for Na^+^ uptake in zebrafish. However, a recent study by Kumai and Perry (2011) did not observe a significant effect of EIPA on Na^+^ influx in zebrafish larvae, bringing the role of NHE3b into question. Further investigation is required to reconcile the inconsistencies between these pharmacological studies. Taking an alternate approach, Shih et al. [29] adopted non-invasive electrophysiological methods to detect Na^+^ uptake activity in intact zebrafish larvae, and observed a decrease in Na^+^ uptake after EIPA treatment or *nhe3b* knockdown. The present *nhe3b* knockdown experiments resulted in a severe decrease in Na^+^ content in zebrafish morphants (Figure 3), further reinforcing the aforementioned perception of NHE3b as a major transporter for Na^+^ absorption. Compared to NHE3, NCC plays a relatively subtle and regulatory role in maintaining Na^+^ and fluid volume homeostasis in mammals [10,11,37]. In zebrafish, treatment with metolazone (an NCC inhibitor) was found to suppress Na^+^ uptake [24]. Besides, sodium green specifically accumulated in HR cells, but not NCC cells [21]. These suggest that zebrafish ionocytes resemble their equivalents in the mammalian kidney, in that NHE3b is the dominant transport protein for maintaining Na^+^ homeostasis, whereas NCC plays a subtle role. This conclusion is further supported by the present study; NHE3b loss-of-function resulted in a drastic reduction in Na^+^ content, but no such effect was observed in the *ncc* morphants (Figures 1, 3). Interestingly, ectoderm derived-zebrafish ionocytes and mesoderm derived-mammalian kidney cells exhibit similar functions and/or regulatory mechanisms, and these physiological processes appear to be conserved during vertebrate evolution from fish to mammals.

In mammals, Na^+^ homeostasis is achieved through compensatory regulation of Na^+^ absorption, which is achieved through differential expression of the relevant Na^+^ transport proteins in the kidneys [9]. Knockout of *nhe3* or *ncc* in mouse resulted in up-regulation of γ-ENaC, to compensate for disruptions in Na^+^ homeostasis [9]. A low salt diet, which impairs body fluid Na^+^ homeostasis, resulted in up-regulated *ncc* expression, but did not affect expression of other Na^+^ transport proteins [3,38]. Here, we knocked down distinct Na^+^ transporters to demonstrate that zebrafish Na^+^ uptake mechanisms exhibit compensatory regulation through differential control of the expression and function of the relevant Na^+^ transport proteins, a process analogous, in terms of some major transporters, to that of Na^+^ reabsorption in the mammalian nephron. Knockdown of *gcm2* (encoding a transcription factor which positively regulates HR cell differentiation) results in loss of NHE3b-expressing HR cells [32,33] and an increase in NCC cell number [39] (Figure 2) in zebrafish larvae. Similarly, loss-of-function of NHE3b induced an increase of NCC cell number (Figure 3). On the other hand, the number of HR cells was found to increase in *ncc* morphants (Figure 1). Differential regulation of larval HR and NCC cell densities was accompanied by changes in Na^+^ content or influx, reflecting disruptions in Na^+^ uptake function (Figures 1, 2 and 3) (and thus the body fluid Na^+^ homeostasis) and subsequent homeostatic adjustment. Taken together, we propose that if body fluid Na^+^ homeostasis is disturbed by decreased expression/function of either NHE3b or NCC, this will stimulate a compensatory increase in the expression/function of the other protein, thereby restoring equilibrium.

Compensatory regulation of NHE3b and NCC in zebrafish Na^+^ homeostasis appears to be of physiological significance, based on analyses of these transport pathways during acclimation to environmental stress, such as acidic FW, which is known to disturb Na^+^ homeostasis in aquatic animals. In a recent study, recovery of Na^+^ content and increased Na^+^ uptake were observed in acid-acclimated zebrafish, and NHE3 was suggested to play a major role in this compensatory process, based on pharmacological and loss-of-function experiments [27]; however, no data on NHE3b expression was available to support this conclusion. On the other hand, an earlier study concluded that zebrafish *nhe3b* expression may be down-regulated because the H^+^ gradient is not favorable for the operation of electronetural NHE3b [23]. This raises the possibility that Na^+^ transport proteins other than NHE3b are critical for Na^+^ homeostasis in acid-acclimated zebrafish. The present study has demonstrated that NCC is required for this regulatory mechanism of Na^+^ homeostasis under acid stress. Down-regulation of NHE3b and concomitant up-regulation of NCC were observed not only at the transcription level, but also in terms of cell number (Figures 4, 5 and 6). Notably, the timing of *ncc* mRNA up-regulation is consistent with that of Na^+^ uptake recovery upon acid acclimation (Figure 6), indicating that NCC, instead of NHE3b, is the major participant in this compensatory process. As such, the two Na^+^ transporters, NHE3b and NCC, in the skin/gills of zebrafish have functional redundancy in regulating Na^+^ absorption. In a similar manner to acid-acclimated larvae, *nhe3b* morphants exhibited an initial decrease in Na^+^ content at 2 dpf, and a subsequent partial recovery at 4 dpf, accompanied by an increase in NCC cell number (Figures 3, 6). This reinforces our conclusion that the initial loss of Na^+^ in acid-treated larvae results from down-regulation of NHE3b, and the impaired Na^+^ content and/or decreased level of NHE3b may trigger an increase in NCC cell number and the concomitant compensatory recovery in Na^+^ content.

Na^+^ content reflects changes in both influx and efflux. One may consider passive Na^+^ efflux as one regulatory process for body fluid Na^+^ homeostasis; however, it appears not to be the case in acid-acclimated zebrafish. In a recent study on the Na^+^ content and fluxes in acid-acclimated zebrafish, the recovery of Na^+^ content was primarily resulted from regulating Na^+^ uptake, rather than Na^+^ efflux [31]. Since NHE3b (Slc9a3b) and NCC (Slc12a10.2) are specifically expressed in the respective HR and NCC ionocytes in zebrafish skin and gills [17,23,24], the differential regulations of the 2 transporters found in the present study (see above) appear to reflect the majority of the Na^+^ uptake changes associated with the compensatory recovery in Na^+^ content. Subsequent loss-of-function experiments further reinforce the physiological significance of the differential regulations of the 2 transporters in body fluid Na^+^ homeostasis during acid acclimation. Knockdown of *ncc* did not significantly affect larval Na^+^ content, but the same treatment under acid stress resulted in a severe reduction of Na^+^ content as compared to wild type (Figure 7). This indicates that NCC plays a minor or subtle role in normal FW conditions, but replaces attenuated NHE3b as the major player in zebrafish Na^+^ homeostasis during acclimation to an acidic environment.

Teleost gill (or skin during the embryonic stages) is an organ with physiological plasticity, being able to modify its morphology and functions to cope with environmental changes. Modifications of the gills are stress- and species- dependent, and include alterations in the size, number, expression pattern, and apical morphology of ionocytes and the lamellar surface area [12,14,40-42]. The ionocytes that appear in the gill lamella were thought to migrate from the gill filaments, and this was suggested as a regulatory mechanism in response to environmental changes [12,43]. Such migration of ionocytes is now thought to be unlikely, as ionocyte progenitor cells were recently observed in not only the filament, but also the lamellar epithelia in zebrafish gills [44]. Different types of ionocyte differentiate from the same pool of epithelial stem cells [34,35]. Acid acclimation was found to increase proliferating stem cells in zebrafish larval skin [45], followed by a resulting increase in the cell densities of all ionocyte types [45] (Figures 4, 5 and 6). The increase in ionocytes may have led to these cells competing for the limited surface area in the larval skin (head, yolk and trunk) or adult gills (filaments and lamellae). The present observations in both the larval skin and adult gills of zebrafish provide explicit evidence to support this concept. The increase in NCC cells in acid-acclimated larvae was restricted to the head region, with no increase observed in the yolk or trunk regions (Figure 6). Generally, NaR and HR cells are predominantly localized to the yolk and the skin of the trunk in organisms in normal fresh water [34]; under acidic conditions, NaR and HR cells increase and cover most of the surface area of these regions [45], thereby occluding NCC cells (Figure 6). The hypothesis that HR and NCC cells compete for skin surface area is also supported by our loss-of-function experiments. Loss-of-function of Gcm2 resulted in a 2–2.7 fold increase in NCC cell density throughout larval skin (Figure 2), while the additional NCC cells were mainly confined to the head region (but not the trunk region) in *nhe3b* morphants (Figure 3). This subtle difference may be because HR cells with attenuated *nhe3b* expression continued to occupy the surface of the trunk region in the *nhe3b* morphants. In the case of adult gills, NCC cell density in the filaments, where all ionocyte types primarily localize in normal FW, was unaffected by acid acclimation (Figure 5), on account of competition with increased HR and NaR cells at these regions (Figure 4); however, the total number of NCC cells in acid-acclimated gills increased due to the additional NCC cells that extended to the lamellae (Figure 5) [32]. In addition, the width of gill filaments increased after acid acclimation (Figures 4, 5), and the resulting increase in gill surface area may have provided more space for ionocyte proliferation.

Cell differentiation is considered to be an important strategy for maintaining body fluid homeostasis, and it appears to be conserved from zebrafish to mammals. One example is the intercalated cells that regulate H^+^ and HCO_3_^-^ transport in the mammalian kidney. Changes in extracellular pH directly induce conversion of β intercalated cells to α intercalated cells, through hensin (DMBT1)-mediated cell differentiation [46,47]. A similar response was also observed in zebrafish, which regulate the proliferation and differentiation of ionocytes in the skin and gills. Hypothermia is known to increase ionocyte number (and thus ionocyte function), through extending cell lifespan by reducing the cell turnover rate and simultaneously triggering cell differentiation of pre-existing undifferentiated progenitors [44]. On the other hand, acidic environments activate proliferation of epidermal stem cells, resulting in an increase of mature ionocytes [32,45]. The present finding that increased NCC cell number is accompanied by a recovery of Na^+^ content suggests that stress-induced NCC cell differentiation is associated with functional regulation of Na^+^ uptake. Regulation of the differentiation of NaR and HR cells depends on the differential expression of Foxi3a and Foxi3b [21,34,35], and Gcm2 is specifically required for the differentiation of HR cells [32,33]. This study has further demonstrated that Foxi3a and -3b are also required for NCC cell differentiation (Additional file 1: Figure S1), while Gcm2 may negatively regulate this process. As described above, both acid acclimation and Gcm2 loss-of-function resulted in an increase of NCC cells. Notably, the newly-recruited NCC cells were confined to the head region (where HR cells do not appear) in acid-acclimated larvae with a concomitant increase of *gcm2* expression, while in *gcm2* morphants, the additional NCC cells occupied the yolk/yolk extension (to which HR cells are normally localized). Taken together, it seems that HR and NCC cells may originate from the same precursor pool, and cell fate may be determined by Gcm2. Under acid stress, up-regulation of *gcm2* triggers HR cell differentiation in the yolk/yolk extension [32], while the ionocyte progenitors in the head region may receive signals other than Gcm2 to differentiate into NCC cells. Further studies are required to determine the identity of such signals, and to investigate differences in the level of the Gcm2 signal between the yolk sac and head regions in zebrafish larvae under acidic stress.

## Conclusions

NHE3b-expressing HR cells and NCC-expressing NCC cells in zebrafish skin/gills are analogous to the proximal tubular cells and distal convoluted tubular cells in mammalian kidneys, respectively, in terms of ion transporter expression and Na^+^ absorption function [15]. This evolutionary conservation from zebrafish to mammals appears to extend to the functional regulation of those ionocytes. Expression and function of the two types of ionocyte and their major transporters, NHE3b and NCC, are differentially regulated, in a mutual compensatory pattern, to cope with physiological or environmental stresses that disturb body fluid Na^+^ homeostasis. These findings provide new insights into this process in fish as well as in mammals. Although much remains to be studied in the future, zebrafish, with its advantages in the availability of molecular/cellular/physiological approaches, is an emerging and competent model for *in vivo* studies of the functional regulation of epithelia transport for body fluid homeostasis.

## Materials and methods

### Animals

The wild type AB strain of zebrafish (*Danio rerio*) was obtained from stocks held at the Institute of Cellular and Organismic Biology, Academia Sinica. Zebrafish were kept in an aquatic tank at 28.5°C under a photoperiod of 14 h of light/10 h of darkness. Fertilized eggs were collected from mating pairs within 30 min after fertilization, and were incubated in local tap water (FW) at 28.5°C. The major ion concentrations of FW are: [Na^+^], 0.5 mM; [K^+^], 0.3 mM; [Ca^2+^], 0.2 mM; [Mg^2+^], 0.16 mM; and [Cl^-^], 0.5 mM. Embryos were collected within 30 min after fertilization, and incubated in a Petri dish until the desired developmental stage. The experimental protocols were approved by the Academia Sinica Institutional Animal Care and Utilization Committee (approval no.: RFiZOOHP2010081).

### Acclimation experiments

FW (control, pH 7.1 ~ 7.4) and acidic FW (pH 4.00 ~ 4.05) were prepared to determine the effects of an acidic medium, following previously described methods [23]. The acidic medium was made by adding H_2_SO_4_ to FW; the concentrations of other ions in acidic FW were the same as those in normal FW. Adult zebrafish were acclimated for 7 d to either acidic FW or FW, and all showed normal swimming behavior with no mortality during the acclimation period. During the experiments, prepared acidic FW stock was continuously pumped into the experimental tank bottom with an electric pump to maintain a stable pH. The embryos were transferred to the Petri dishes containing FW or acidic FW immediately after fertilization, and were incubated for up to 5 d with daily renewal of the media. Temperature, pH, and the light–dark cycle were controlled within a stable range. The ion concentrations were determined by atomic absorption spectrometry (U-2000, Hitachi, Tokyo, Japan). Gills and larvae were sampled and/or fixed for subsequent analysis at different times post acclimation, depending on the experiment.

### Quantitative real-time PCR (qPCR)

Expression levels of target gene mRNA were measured by qRT-PCR with a LightCycler real-time PCR system (Roche, Penzberg, Germany). Each PCR consisted of 30 ng of cDNA, 500 nM of each primer, and LightCycler 480 SYBR Green Master (Roche Applied System) in a final volume of 10 μl. The primer sets for qPCR analysis were as follows: *nhe3b* (F 5′-GCGAAACCCACCCTGGCAAAC-3′, R 5′-GGCGAAGGAGTCTGTGGAGCG-3′); *ncc* (F 5′-GACCCAAGGTGGAGAGGACG-3′, R 5′-CAGTTGATACCGATACTCAGC-3′). PCR products were subjected to a melting-curve analysis, and representative samples were electrophoresed to verify that only a single product was present. Control reactions were conducted with sterile water to determine background and genomic DNA contamination levels. The standard curve of each gene was confirmed to be in a linear range with β-actin as an internal control.

### Immunocytochemistry

Samples were fixed with 4% paraformaldehyde in phosphate-buffered saline (PBS; 1.4 mM NaCl, 0.2 mM KCl, 0.1 mM Na_2_HPO_4_, and 0.002 mM KH_2_PO_4_; pH 7.4) solution for 1 h at 4°C. After being rinsed with PBS, samples were incubated with 3% bovine serum albumin (BSA) for 1 h to block nonspecific binding. Afterward, samples were incubated with polyclonal antibodies against the A-subunit of zebrafish H^+^-ATPase (HA) (synthetic peptide: AEMPADSGYPAYLGARLA), the C-terminal domain (APHKPNGKPADPNRDH) of zebrafish NHE3b, or the N-terminal domain (IKKSRPSLDVLRNPPDD) of zebrafish NCC-like 2 (*slc12a10.2*) (diluted 1:200; customization produced by Genomics, Taipei, Taiwan), at 4°C overnight. Samples were further incubated with goat anti-rabbit IgG conjugated with Alexa Fluor 488 (Molecular Probes, 1:200 diluted with PBS) at room temperature for 2 h. Observation and image acquisition were carried out using an upright fluorescence microscope (Axioplan 2 Imaging, Carl Zeiss, Oberkochen, Germany). HR cells are mainly distributed at the surface of the yolk and yolk extension, while NCC cells are evenly dispersed over the whole body skin, including the head, trunk, yolk sac, and yolk extension regions in zebrafish larvae under normal FW conditions. For comparison of cell density, the HR and NCC cells in 10–12 unit areas (100 × 100 μm^2^ each) of yolk sacs were counted and averaged for each individual (Additional file 2: Figure S2A). The total number of NCC cells in each region (yolk sac/extension, trunk and head, Additional file 2: Figure S2B) were also counted. HR and NCC cells are mainly distributed in the gill filaments of adult zebrafish gills under the normal FW condition. The middle parts within second pairs of the adult gills were sampled for immunostaining, and NCC cells within a given length (250 μm in the distal edge of the filament) of two randomly-selected filaments for each individual were counted and averaged (Additional file 2: Figure S2C, C-1). Total NCC cell numbers in the selected filaments and lamellae (within the given length 250 μm) were counted and summed together (Additional file 2: Figure S2C-2). The specificities of the antibodies against zebrafish NHE3b and NCC-like 2 were confirmed by western blot analysis (Additional file 3: Figure S3).

### Translational knockdown with antisense morpholino oligomers

Morpholino oligonucleotides (MOs) were obtained from Gene Tools (Philomath, OR, USA). The MOs were as follows: *foxi3a* (+44 to +68, against 5′UTR), 5′-TCTTCCCGTTTCTCTTTGTTGAAGG-3′; *foxi3b* (+64 to +88, against 5′UTR), 5′-CTCGATCCTGAGGGTGCTCCAGTTG-3′; *gcm2* (+320 to +344, against ATG), 5′-AAACTGATCTGAGGATTTGGACATG-3′; *slc12a10.2* (NCC; against 92–116 nucleotides within the coding region) 5′-TTGCCAAAATCAGCCTCTCCCATAT-3′; and *slc9a3b* (NHE3b; +90 to +114 against ATG), 5′-AGTAGAAAACGCCATATCAGAACGC-3′. The MOs were diluted with 1X Danieau injection buffer (0.4 mM MgSO_4_; 0.6 mM CaCl_2_; 0.7 mM KCl; 58 mM NaCl; 5 mM Hepes, pH 7.6) and injected using an IM-300 microinjection system (Narishigi Scientific Instrument Laboratory, Tokyo, Japan) into 1 ~ 2-cell-stage embryos at concentrations of 1, 2, or 4 ng/embryo. The maximal concentration that caused no obvious toxic effects on embryogenesis was 2 ng/embryo, and this was thus used in the following experiments. Phenol red at 0.1% was used as a visualizing indicator. Most of the morphants had normal size as the wild type embryos. Few morphants that had abnormal phenotype were excluded from following studies. The specificities and effectiveness of *ncc* and *nhe3b* morpholinos were confirmed by western blot analysis (Additional file 3: Figure S3).

### Western blot analysis

Forty larvae were pooled and homogenized as one sample. Protein samples were separated via 10% SDS-PAGE electrophoresis as the amount 40 μg/well. After transferred the proteins onto the polyvinylidene difluoride membrane (Millipore), the blots were incubated with antibodies against NHE3b and NCC-like 2 diluted 1:450 and 1:800, respectively. The membranes were further incubated with an alkaline-phosphatase-conjugated goat anti-rabbit IgG (dilute 1:2500, Jackson Laboratories) and then developed with 5-bromo-4-chloro-3-indolyphosphate/nitro- blue tetrazolium. Antibody that against β-actin was used as internal control.

### ^24^Na^+^ influx analysis

Tracer media were prepared by adding appropriate amounts of ^24^NaHCO_3_ (prepared in a 1-mW Tsing Hua Open-Pool Reactor, Nuclear Science and Technology Department Center, National Tsing Hua University, Hsinchu, Taiwan) to normal FW to give a final working specific activity of 20,000-36,000 cpm.mol^-1^. Fifteen larvae (one group) were transferred to the tracer medium, and incubated for 4 h. After incubation, larvae were washed several times with isotope-free fresh water. Fifteen larvae were pooled to form one sample, and the ^24^Na^+^ absorbed within the sample was analyzed using a gamma counter (model B5002; Packard, Meriden, CT). Unidirectional Na^+^ influx was calculated using the following formula: *J*_in_ = Q_larva_.*X*^-1^_out_.*t*^-1^, where *J*_in_ is the influx (pmol.larva^-1^.h^-1^), Q_larva_ is the radioactivity of the larva (CPM/individual) at the end of the incubation, *X*_out_ is the specific activity of the incubation medium (cpm / pmol), and *t* is the incubation time (h).

### Detection of whole body Na^+^ content

Twenty zebrafish larvae were pooled as one sample and subjected to digestion with 13.1 N HNO_3_ at 60°C overnight. Digested solutions were diluted with double-deionized water, and the total Na^+^ content was measured with an atomic absorption spectrophotometer. Sodium standard solution (Merck, Darmstadt, Germany) was used to mark the standard curves.

### Statistical analysis

Values were presented as the mean ± standard deviation (SD), and were compared using Student’s *t*-test and/or one-way analysis of variance (ANOVA).

## Competing interests

The authors declare that they have no competing interests.

## Authors’ contributions

WJC and YFW conceived of the study. WJC and PPH drafted the manuscript. WJC designed the experiments, collected data, and analyzed the results. HJH assisted in quantitative PCR assay and statistical analysis. JHW contributed to immunostaining and image collection. THL and PPH supervised the experiments. All authors read and approved the final manuscript.

## Supplementary Material

Additional file 1: Figure S1 Double knockdown of *foxi3a* and *foxi3b* blocks NCC cell differentiation in 3-dpf morphants (MO). Arrows indicate immunostaining for NCC in wild type embryos (WT). Scale bar = 100 μm. Click here for file

Additional file 2: Figure S2 Sampling and quantifying methods for cell number and cell density. *A*: The 12 selected areas for measuring NCC and HR cell densities in the embryonic yolk sac surface. Unit area: 100 × 100 μm^2^. *B*: Regions of embryonic skin: head (I), yolk (II), and trunk (III). Scale bar = 100 μm. *C*: The middle part of 2^nd^ gill arch for sampling and immunostaining. *C-1,* Areas of the gill filament (250 μm in total length) selected for cell density measurements. *C-2,* The area (filament and lamella within 250 μm in length) selected for determining total cell number. Click here for file

Additional file 3: Figure S3 Specificity and effectiveness of *ncc* and *nhe3b* morpholinos. Western blot analysis was conducted to detect the protein expression of NHE (*A*) and NCC (*B*). Three-dpf wild type larvae (WT), *nhe3b* morphants (NHE MO), and *ncc* morphants (NCC MO) were sampled for the analysis. Arrows indicate the protein with the predicted size. β-actin was used as internal control. Click here for file
