# Supplementary figures and images for: Compensatory regulation of Na+ absorption by Na+/H+ exchanger and Na+-Cl- cotransporter in zebrafish (Danio rerio)
Source: Front Zool. 2013 Aug 7;10:46. doi: 10.1186/1742-9994-10-46 (PMC3750650; doi:10.1186/1742-9994-10-46)

## Slide 1
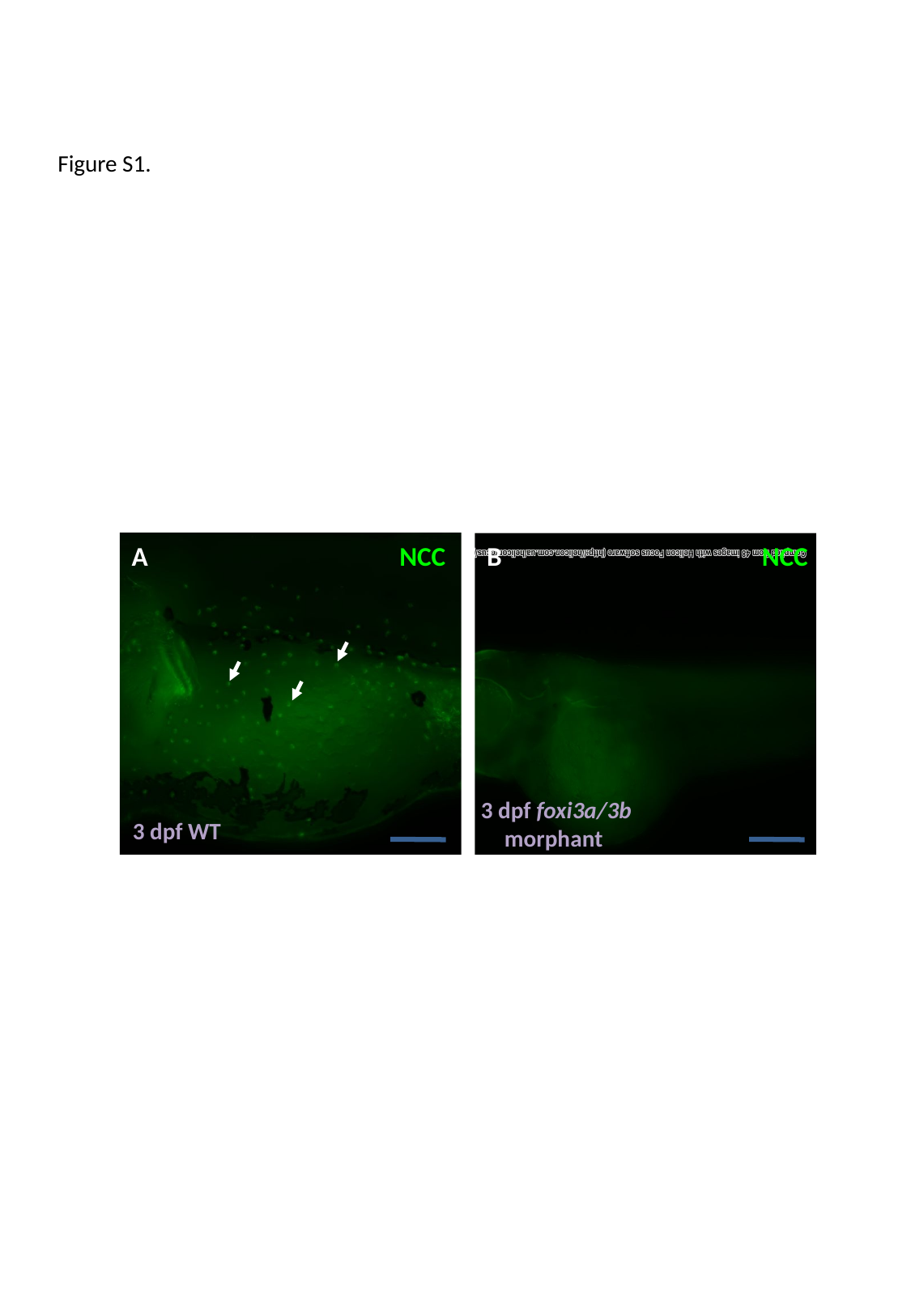

# Figure S1.
A
NCC
B
NCC
3 dpf foxi3a/3b morphant
3 dpf WT

Supplement: Additional file 1: Figure S1 — Double knockdown of foxi3a and foxi3b blocks NCC cell differentiation in 3-dpf morphants (MO). Arrows indicate immunostaining for NCC in wild type embryos (WT). Scale bar = 100 μm. [file 1742-9994-10-46-S1.pptx]

## Slide 1
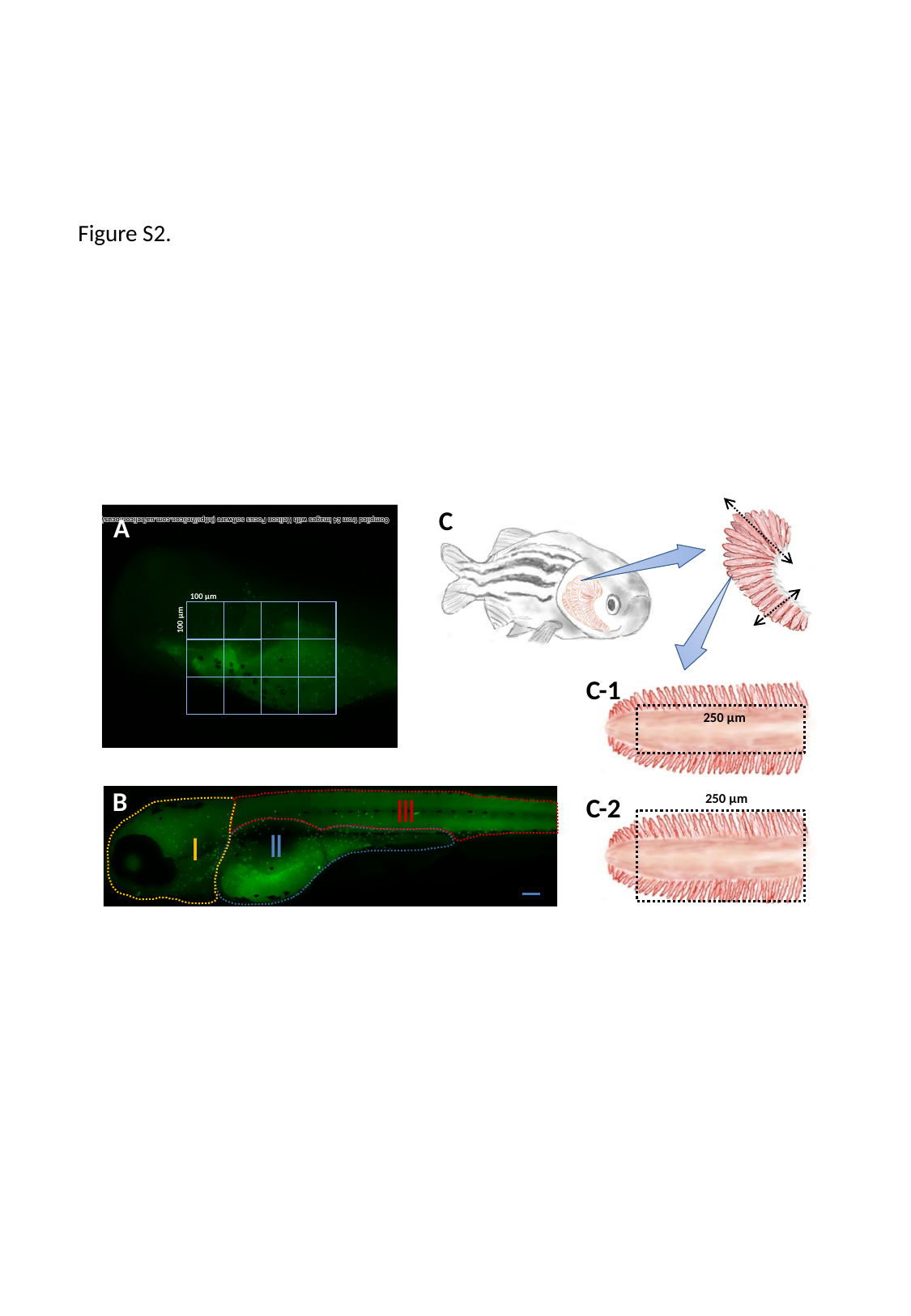

Figure S2.
C
A
100 μm
100 μm
C-1
250 μm
B
Ⅲ
Ⅱ
Ⅰ
250 μm
C-2

Supplement: Additional file 2: Figure S2 — Sampling and quantifying methods for cell number and cell density. A: The 12 selected areas for measuring NCC and HR cell densities in the embryonic yolk sac surface. Unit area: 100 × 100 μm2. B: Regions of embryonic skin: head (I), yolk (II), and trunk (III). Scale bar = 100 μm. C: The middle part of 2nd gill arch for sampling and immunostaining. C-1, Areas of the gill filament (250 μm in total length) selected for cell density measurements. C-2, The area (filament and lamella within 250 μm in length) selected for determining total cell number. [file 1742-9994-10-46-S2.pptx]

## Slide 1
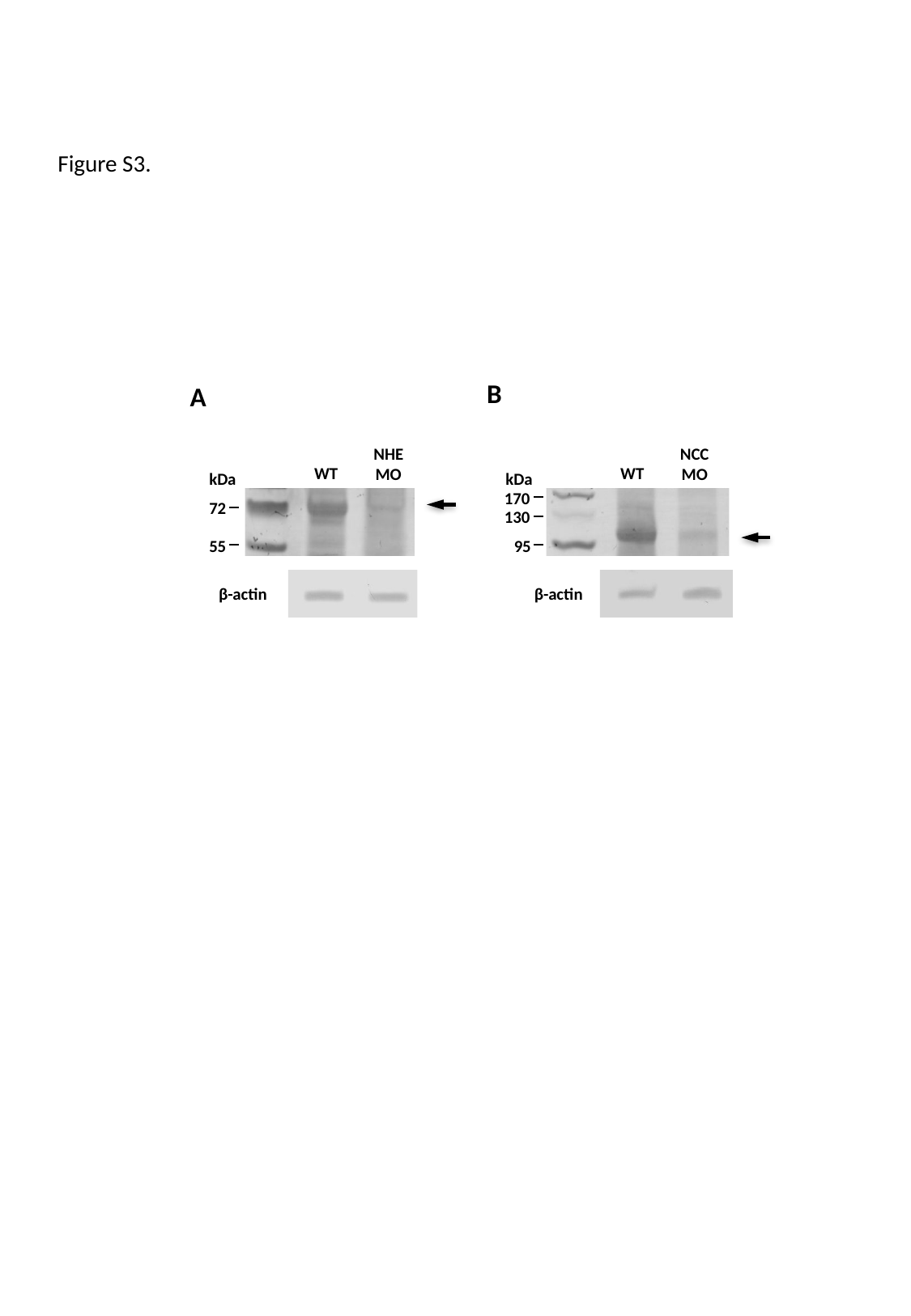

#
Figure S3.
B
A
NHE MO
NCC MO
WT
WT
kDa
kDa
170
72
130
55
95
β-actin
β-actin

Supplement: Additional file 3: Figure S3 — Specificity and effectiveness of ncc and nhe3b morpholinos. Western blot analysis was conducted to detect the protein expression of NHE (A) and NCC (B). Three-dpf wild type larvae (WT), nhe3b morphants (NHE MO), and ncc morphants (NCC MO) were sampled for the analysis. Arrows indicate the protein with the predicted size. β-actin was used as internal control. [file 1742-9994-10-46-S3.pptx]
